# Supplementary material for: Disease-specific B cell clones are shared between patients with Crohn’s disease
Source: Nat Commun. 2025 Apr 17;16:3689. doi: 10.1038/s41467-025-58977-y (PMC12006383; doi:10.1038/s41467-025-58977-y)
Supplement: Supplementary file 1 — Supplementary Information [file 41467_2025_58977_MOESM1_ESM.pdf]

Disease-specific B cell clones are shared between patients with Crohn's disease

Prasanti Kotagiri, William M. Rae, Laura Bergamaschi, Diana Pombal, Ji-Yeun Lee, Nurulamin M. Noor, Raoul S. Sojwal, Samuel J. S. Rubin, Lukas W. Unger, Sofie H. Tolmeijer, Giulia Manferrari, Rachael J. M. Bashford-Rogers, David B. Bingham, Anton Stift, Stephan Rogalla, John Gubatan, James C. Lee, Kenneth G. C. Smith, Eoin F. McKinney, Scott D. Boyd, Paul A. Lyons

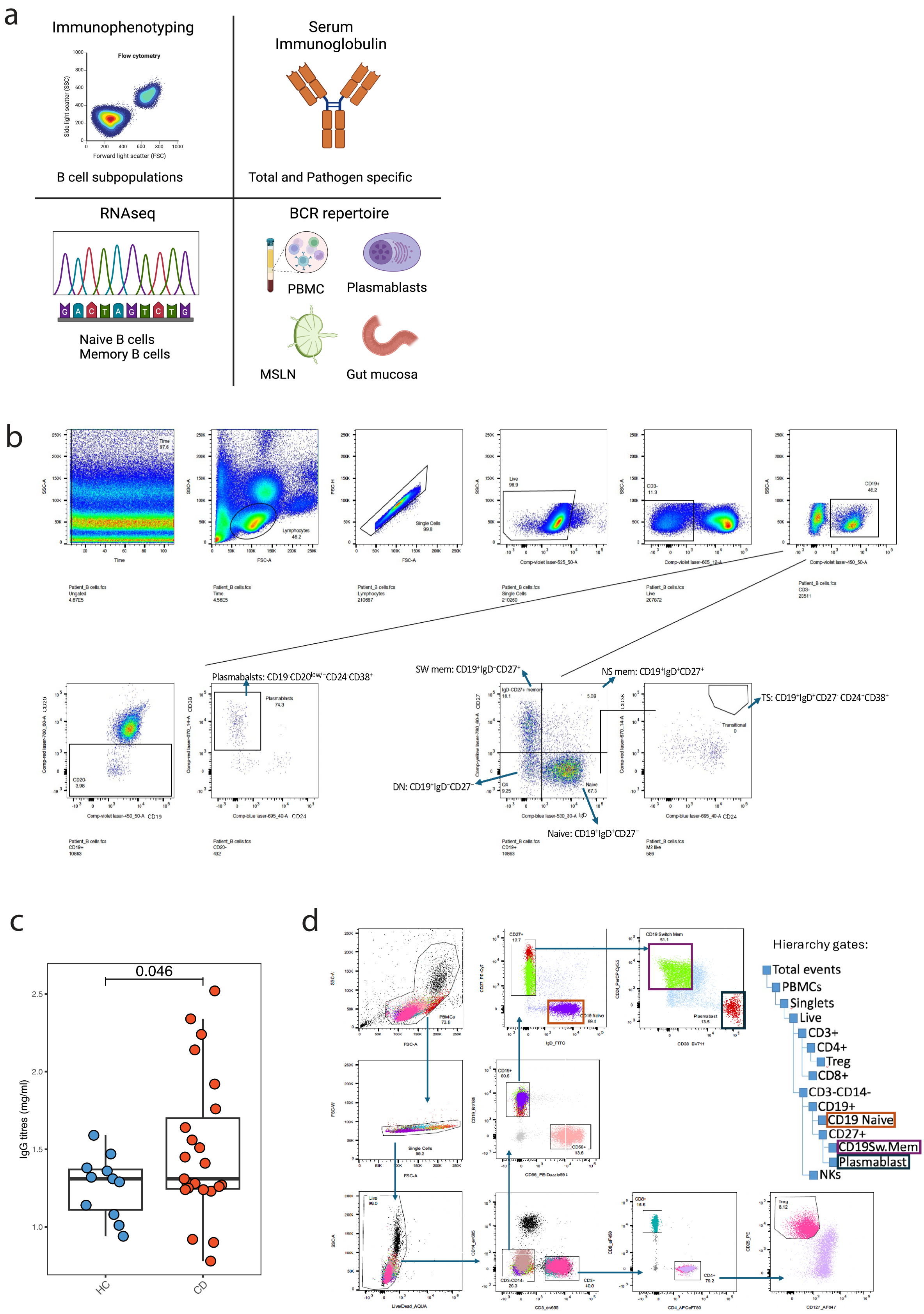
a
Immunophenotyping
Flow cytometry
Side light scatter (SSC)
Forward light scatter (FSC)
B cell subpopulations
Serum
Immunoglobulin
Total and Pathogen specific
RNAseq
Naive B cells
Memory B cells
BCR repertoire
PBMC
Plasmablasts
MSLN
Gut mucosa
b
Plasmablasts: CD19⁻CD20low/−CD24⁻CD38⁺
SW mem: CD19⁺IgD⁻CD27⁺
NS mem: CD19⁺IgD⁺CD27⁺
TS: CD19⁺IgD⁺CD27⁻CD24⁺CD38⁺
DN: CD19⁺IgD⁻CD27⁻
Naive: CD19⁺IgD⁺CD27⁻
c
0.046
IgG titres (mg/ml)
HC
CD
d
Hierarchy gates:
Total events
PBMCs
Singlets
Live
CD3+
CD4+
Treg
CD8+
CD3-CD14-
CD19+
CD19 Naive
CD27+
CD19Sw.Mem
Plasmablast
NKs

Supplementary Figure. 1: B cell immunophenotyping and serology

a. Schematic of methodological workflow. Peripheral blood mononuclear cells (PBMC), mesenteric lymph nodes (MSLN). Created in BioRender. Kotagiri, P. (2024) BioRender.com/h45f791
b. Gating strategy used in immunophenotyping. Gating strategy of peripheral B cells used in immunophenotyping (Figure 1a).
c. Immunoglobulin titres. Boxplot of immunoglobulin titres split according to disease. Immunoglobulin titres in healthy individuals (n = 11), patients with CD (n = 23). Unpaired two-sided t-test. HC coloured in blue and CD coloured in red.
d. Gating strategy used in transcriptomics and BCR repertoire. Gating strategy of peripheral B cells used in generating B cell subset populations for downstream bulk RNAseq and BCR repertoire (Figure 1c-g, Figure 2g-h and Supplementary Figure. 5a-b).

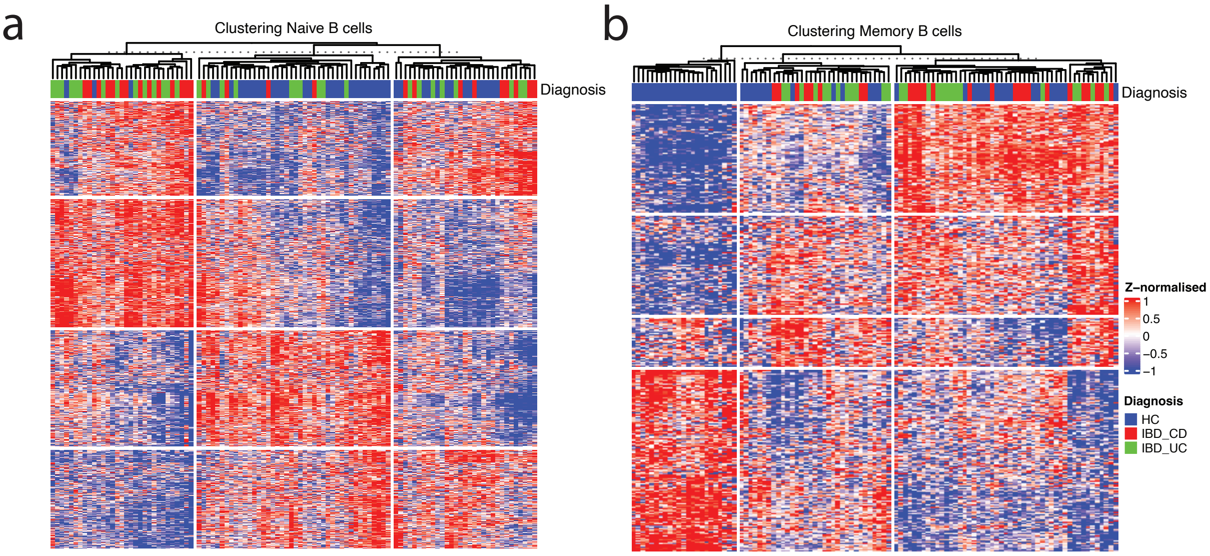

Supplementary Figure. 2: Clustering of differentially expressed genes

a. Naive B cell transcriptome. Unsupervised clustering clustering of Naive B cell differentially expressed genes by Euclidean distance and K-means clustering.
b. Memory B cell transcriptome. Unsupervised clustering clustering of Memory B cell differentially expressed genes by Euclidean distance and K-means clustering.
For **d-e**, naïve B cells (HC= 49, CD=24 and UC=32) and memory B cells (HC= 54, CD=26 and UC=26). Box plots show the median (center line) and interquartile range (25th–75th percentile of the data) and whiskers indicate range of data 1.5 times the interquartile below and above the 25th and 75th percentile, respectively.

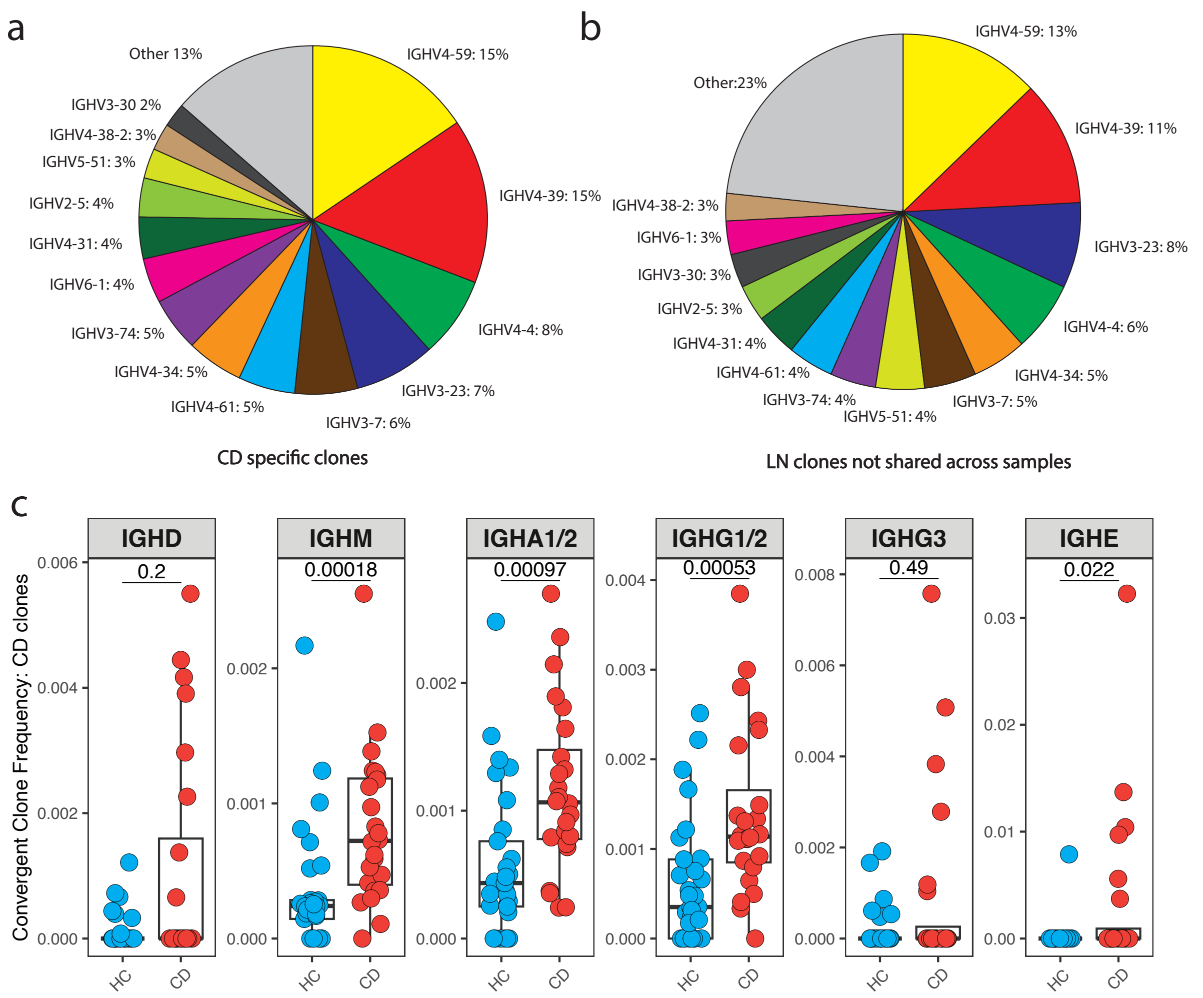

Supplementary Figure. 3: V gene distribution of CD-associated and non-associated clones in MSLN

a. V gene usage in CD-associated clones. Pie chart of V gene usage of CD-associated clones in LN.
b. V gene usage of non-shared clones. Pie chart of V gene usage of LN-clones not shared amongst LN samples.
c. Validation of CD-associated clones per isotype. Assessed clonal convergence of CD-associated clones derived from LN in PBMCs. Boxplot representing convergence split according to sub-isotype and disease. Each dot represents a sample. Unpaired one-sided Wilcoxon's signed-rank test. Healthy individuals (n =29) and CD (n = 24). Box plots show the median (center line) and interquartile range (25th–75th percentile of the data) and whiskers indicate range of data 1.5 times the interquartile below and above the 25th and 75th percentile, respectively.

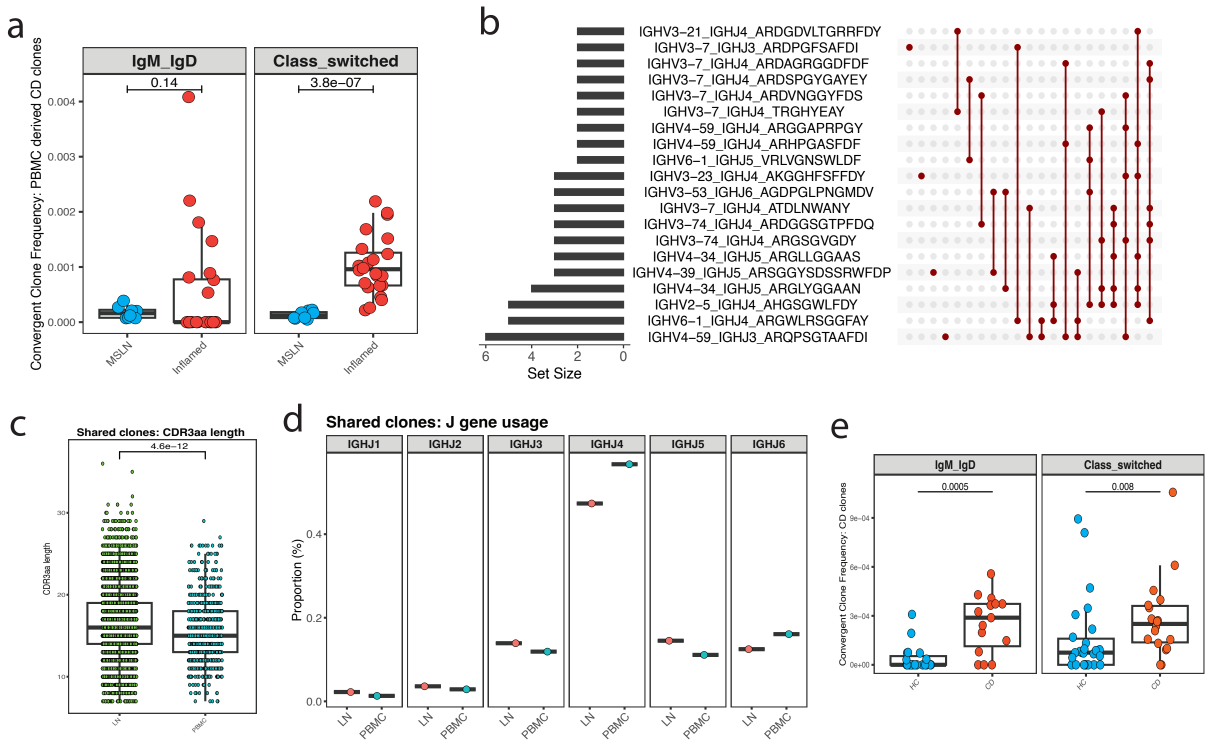

Supplementary Figure. 4: CD-associated BCR clones can be detected in peripheral blood.

a. CD-associated clones derived from PBMC. CD associated clones were derived based on their presence in PBMCs BCR repertoire in 2 or more CD patients and their absence in health (765 clones). These clones were assessed for enrichment in CD LN and compared to MSLN. Boxplot represents convergence split according to isotype and disease. Each dot represents a sample. Unpaired one-sided Wilcoxon's signed-rank comparing enrichment in post-mortem MSLN and CD LN. MSLNs (n =8) and CD inflamed LN (n = 24).

b. Dominant clones: 20 most frequent PBMC derived CD clones identified in LN. Horizontal bars present the number of PBMC samples a given clone is present in. Lines connecting dots on the right illustrate the pattern of co-existence of multiple of clones in a given sample. The dot symbolizes the presence of the clone in the combination.

c. CDR3 length: Comparison of CDR3 amino acid lengths of CD-associated BCR clones derived from CD LN versus CD PBMC. Each dot represents a clone. Unpaired two-tailed t test. CD LN (n =24) and CD PBMC (n = 24).

d. J gene usage: Dot plot representing the J gene proportional use of CD-associated BCR clones derived from CD LN versus CD PBMC. CD LN (n =24) and CD PBMC LN (n = 24).

e. Validation of CD-associated LN clones in PBMCs: Boxplots of enrichment of a refined list of CD-associated LN clones. Each dot represents a sample. One-sided unpaired Wilcoxon's signed-rank test. CD (n =24) and HC (n = 29).

Box plots show the median (center line) and interquartile range (25th–75th percentile of the data) and whiskers indicate range of data 1.5 times the interquartile below and above the 25th and 75th percentile, respectively.

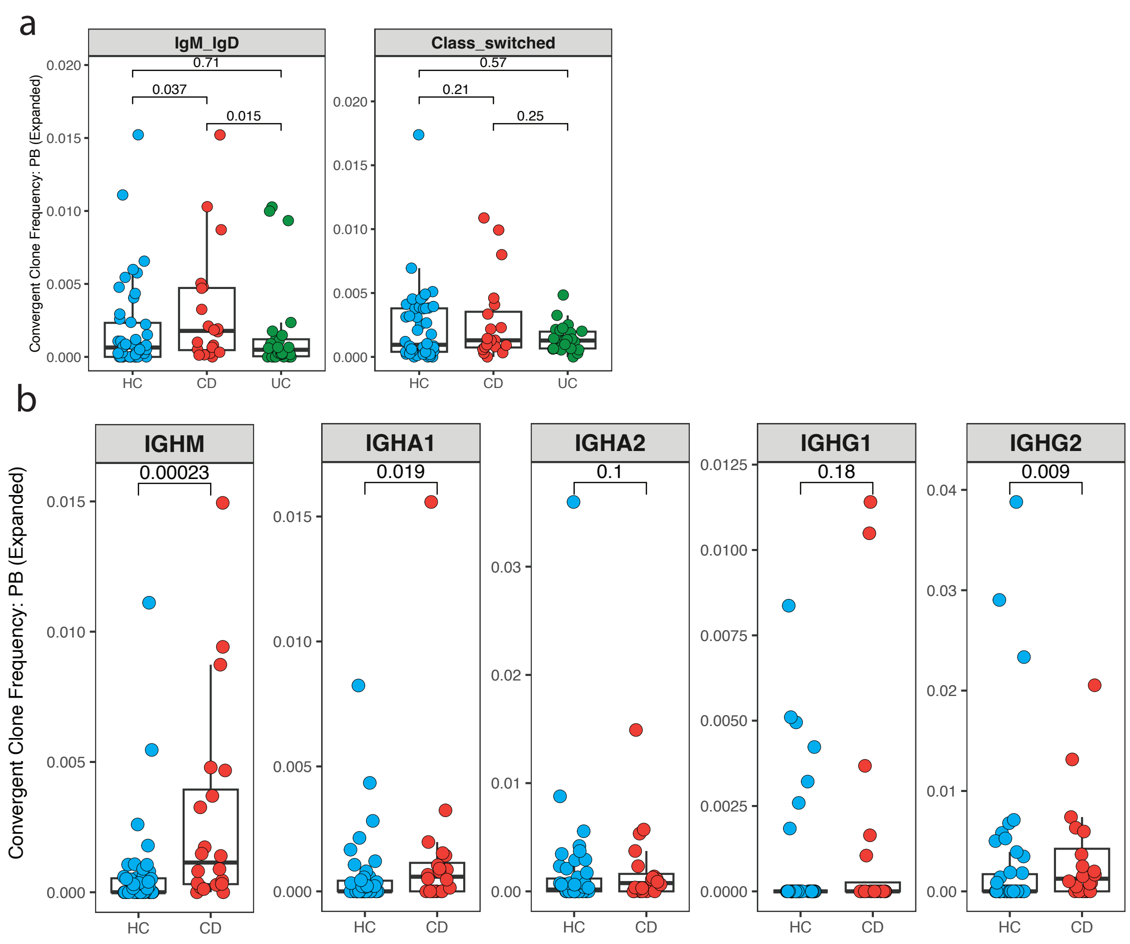

Supplementary Figure. 5: CD-associated BCR clones can be detected in peripheral blood.

a. Validation of CD-associated LN clones in Plasmablasts: Boxplots of enrichment of CD-associated LN clones in plasmablasts according to unique UMI and not unique clone thus taking clonal expansion into consideration. Each dot represents a sample. One-sided unpaired Wilcoxon's signed-rank test.

b. Validation of CD-associated clones per isotype: Assessed clonal convergence of CD-associated clones derived from LN in plasmablasts according to unique UMI. Boxplot representing convergence split according to sub-isotype and disease. Each dot represents a sample. One-sided unpaired Wilcoxon's signed-rank test.

For **a-b**, n = 46 for healthy individuals and n = 20 and n = 26for patients with CD and UC respectively. Box plots show the median (center line) and interquartile range (25th–75th percentile of the data) and whiskers indicate range of data 1.5 times the interquartile below and above the 25th and 75th percentile, respectively.

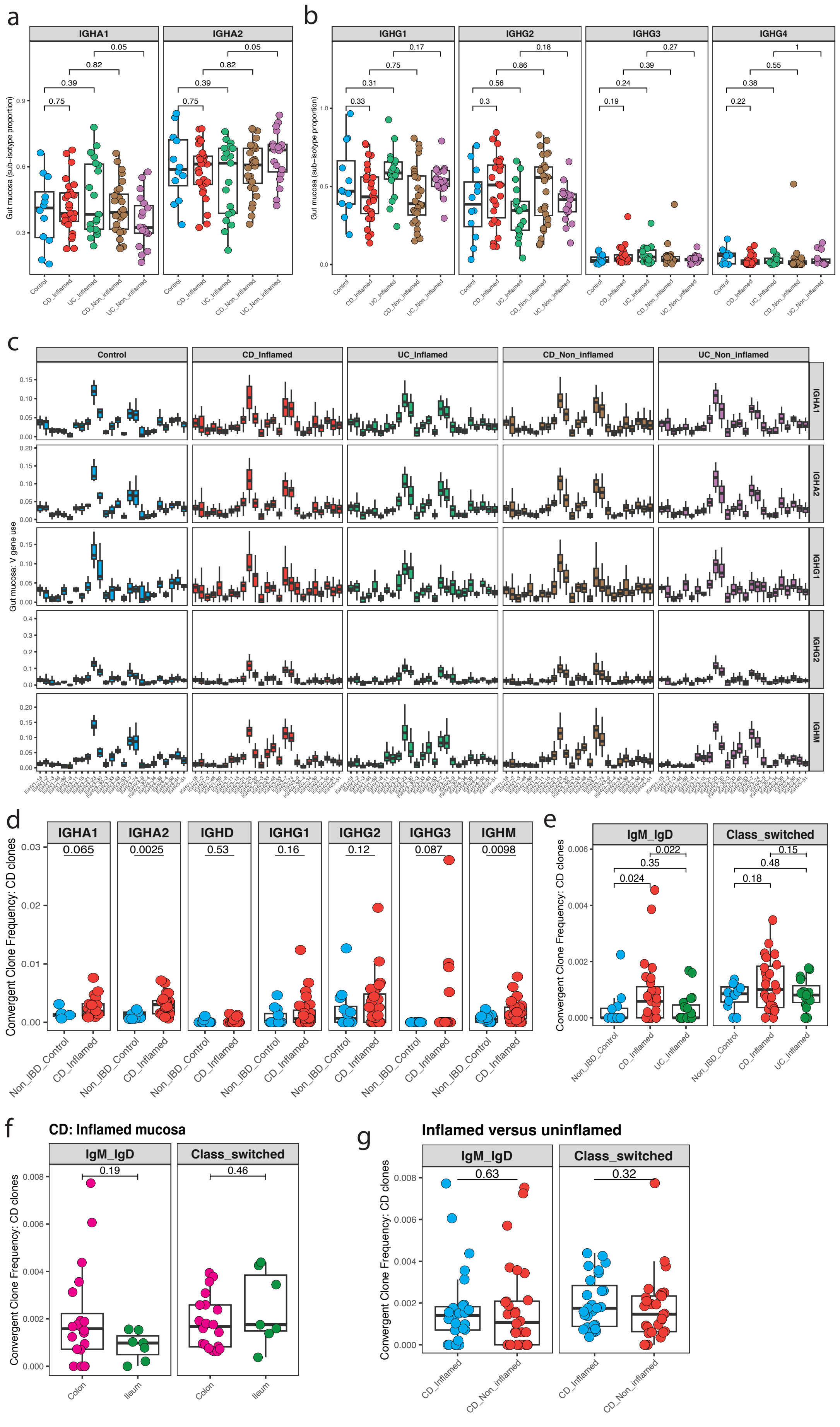

a
IGHA1
IGHA2
Gut mucosa (sub-isotype proportion)
b
IGHG1
IGHG2
IGHG3
IGHG4
c
Control
CD_Inflamed
UC_Inflamed
CD_Non_inflamed
UC_Non_inflamed
Gut mucosa: V gene use
d
IGHA1
IGHA2
IGHD
IGHG1
IGHG2
IGHG3
IGHM
Convergent Clone Frequency: CD clones
e
IgM_IgD
Class_switched
f
CD: Inflamed mucosa
Colon
Ileum
g
Inflamed versus uninflamed

Supplementary Figure. 6: Intestinal BCR Repertoire analysis reveals differences in CD compared with health.

a. IGHA Sub-isotype use: Boxplots showing IGHA sub-isotype proportions according to inflammation status. Each dot represents a sample. Unpaired two-sided t test.
b. IGHG Sub-isotype use: Boxplots showing IGHG sub-isotype proportions according to inflammation status. Each dot represents a sample. Unpaired two-sided t test.
c. V gene use: Boxplots showing V gene proportions per sample split according to inflammation status and sub-isotype.
d. Validation of CD-associated clones per isotype: Assessed clonal convergence of CD-associated clones derived from LN in gut mucosa. Boxplot representing convergence split according to sub-isotype and disease. Each dot represents a sample. Unpaired one-sided Wilcoxon's signed-rank test.
e. Validation of refined CD-associated clones: Assessed clonal convergence of refined list of CD-associated clones derived from LN in gut mucosa. Boxplot representing convergence split according to isotype and disease. Each dot represents a sample. Unpaired one-sided Wilcoxon's signed-rank test.
f. Enrichment of CD-associated clones according to disease location: Assessed clonal convergence of CD-associated clones derived from LN in gut mucosa from patients with CD. Boxplot representing convergence split according to isotype and disease location. Each dot represents a sample. Unpaired two-sided Wilcoxon's signed-rank test.
g. Enrichment of CD-associated clones according to inflammation state: Assessed clonal convergence of CD-associated clones derived from LN in gut mucosa from patients with CD. Boxplot representing convergence split according to isotype inflammatory state. Each dot represents a sample. Unpaired two-sided Wilcoxon's signed-rank test.
For **a-g**, Non-IBD control = 12, CD-inflamed = 27, UC-inflamed = 19, CD-uninflamed = 28, UC-uninflamed = 19, CD-inflamed-colon= 20, CD-inflamed-colon= 27. Box plots show the median (center line) and interquartile range (25th–75th percentile of the data) and whiskers indicate range of data 1.5 times the interquartile below and above the 25th and 75th percentile, respectively.

	B cell Flow	B cell RNAseq	Serum Ig	Serum gut pathogen specific antibodies	BCR repertoire: MSLN	BCR repertoire: PBMC	BCR repertoire: Plasmablasts	BCR repertoire: Gut mucosa
Cohort 1								
Cohort 2								
Cohort 3								
Cohort 4								

Table S1. Cohort profiling using different B cell phenotyping methods.

**Marker**	**Manufacturer**	**CAT #**	**Dilution**
CD19	BioLegend	302240	1/40
CD3	eBiosciences	86-0037	1/40
CD38	BioLegend	303528	1/20
CD14	eBiosciences	83-0149	1/20
CD4	BioLegend	47-0049	1/66
CD8	eBiosciences	48-0087	1/40
CD127	BD	558598	1/20
CD25	BioLegend	356103	1/40
CD56	BioLegend	318348	1/40
CD27	eBiosciences	25-0279	1/66
IgD	BD	555778	1/40
CD24	BD	561647	1/66

Table S2. Antibodies used in Flow cytometry.
