# Supplementary material for: Disease-specific B cell clones are shared between patients with Crohn’s disease
Source: Nat Commun. 2025 Apr 17;16:3689. doi: 10.1038/s41467-025-58977-y (PMC12006383; doi:10.1038/s41467-025-58977-y)
Supplement: Supplementary file 2 — Description of Additional Supplementary Files [file 41467_2025_58977_MOESM2_ESM.pdf]

Description of Additional Supplementary Files

Supplementary Data 1. Clinical Data of RNAseq samples

Supplementary Data 2. Differential Gene Expression analysis of Naïve B cells where P values and FDRs are determined by moderated t-tests and Benjamini-Hochberg multiple test correction.

Supplementary Data 3. Differential Gene Expression analysis of Memory B cells where P values and FDRs are determined by moderated t-tests and Benjamini-Hochberg multiple test correction.

Supplementary Data 4. Clinical Data of BCR repertoire samples

Supplementary Data 5. Read counts of BCR repertoire libraries

Supplementary Data 6. Serum antibody pathogen array titres

Supplementary Data 7. Clinical Data of IBD gut mucosa samples

Supplementary Data 8. Differential V gene analysis of gut mucosa where P values and FDRs are determined by t-test and Benjamini-Hochberg multiple test correction.
