# Supplementary material for: Disease-specific B cell clones are shared between patients with Crohn’s disease
Source: Nat Commun. 2025 Apr 17;16:3689. doi: 10.1038/s41467-025-58977-y (PMC12006383; doi:10.1038/s41467-025-58977-y)
Supplement: Supplementary file 11 — Reporting Summary [file 41467_2025_58977_MOESM11_ESM.pdf]

nature portfolio

Corresponding author(s): Prasanti Kotagiri
Paul A Lyons

Last updated by author(s): Sep 18, 2024

Reporting Summary

Nature Portfolio wishes to improve the reproducibility of the work that we publish. This form provides structure for consistency and transparency in reporting. For further information on Nature Portfolio policies, see our Editorial Policies and the Editorial Policy Checklist.

Statistics

For all statistical analyses, confirm that the following items are present in the figure legend, table legend, main text, or Methods section.

n/a	Confirmed	
☐	☒	The exact sample size (*n*) for each experimental group/condition, given as a discrete number and unit of measurement
☐	☒	A statement on whether measurements were taken from distinct samples or whether the same sample was measured repeatedly
☐	☒	The statistical test(s) used AND whether they are one- or two-sided<br>*Only common tests should be described solely by name; describe more complex techniques in the Methods section.*
☐	☒	A description of all covariates tested
☐	☒	A description of any assumptions or corrections, such as tests of normality and adjustment for multiple comparisons
☐	☒	A full description of the statistical parameters including central tendency (e.g. means) or other basic estimates (e.g. regression coefficient) AND variation (e.g. standard deviation) or associated estimates of uncertainty (e.g. confidence intervals)
☐	☒	For null hypothesis testing, the test statistic (e.g. *F*, *t*, *r*) with confidence intervals, effect sizes, degrees of freedom and *P* value noted<br>*Give P values as exact values whenever suitable.*
☒	☐	For Bayesian analysis, information on the choice of priors and Markov chain Monte Carlo settings
☒	☐	For hierarchical and complex designs, identification of the appropriate level for tests and full reporting of outcomes
☒	☐	Estimates of effect sizes (e.g. Cohen's *d*, Pearson's *r*), indicating how they were calculated

Our web collection on statistics for biologists contains articles on many of the points above.

Software and code

Policy information about availability of computer code

Data collection	NA
Data analysis	FlowJo v10.2 for flow cytometry analyses.<br>Imcantation was used for B cell receptor raw reads processing https://immcantation.readthedocs.io/en/stable/index.html<br>IMGT-V QUEST was used for immunoglobulin gene use and sequence annotation<br>IMGT V-QUEST (Lefranc 2011) http://www.imgt.org/HighV-QUEST/<br>B cell receptor repertoire analyses was performed in R version 4.1.0 (2021-05-18)<br><br>R package: stringr version 1.2.0 CRAN https://cran.r-project.org<br>R package: dplyr version 1.0.2 CRAN https://cran.r-project.org<br>R package: tidyr version 1.1.2 CRAN https://cran.r-project.org<br>R package: ggplot2 version 3.3.3 CRAN https://cran.r-project.org<br>R package: EdgeR version 4.0 CRAN https://cran.r-project.org<br>R package: mixOmics_6.18.1<br>R package: limma_3.50.3<br>R package: UpSetR_1.4.0<br>R package: ComplexHeatmap_2.10.0

For manuscripts utilizing custom algorithms or software that are central to the research but not yet described in published literature, software must be made available to editors and reviewers. We strongly encourage code deposition in a community repository (e.g. GitHub). See the Nature Portfolio guidelines for submitting code & software for further information.

Data

Policy information about availability of data

All manuscripts must include a data availability statement. This statement should provide the following information, where applicable:

- Accession codes, unique identifiers, or web links for publicly available datasets
- A description of any restrictions on data availability
- For clinical datasets or third party data, please ensure that the statement adheres to our policy

Sequencing data available from the SRA and EGA

Research involving human participants, their data, or biological material

Policy information about studies with human participants or human data. See also policy information about sex, gender (identity/presentation), and sexual orientation and race, ethnicity and racism.

Reporting on sex and gender	Sex was determined by self reporting. Healthy controls were age and sex matched where possible. The findings of this study does not apply to only one sex.
Reporting on race, ethnicity, or other socially relevant groupings	Race and ethnicity information was not obtained from our participants and not factored in during analysis
Population characteristics	Age, current diagnosis and (where applicable) treatment.
Recruitment	Healthy participants<br>Inclusion criteria for healthy individuals were people aged between 20-77 years, with no serious co-morbidities, no direct family history of autoimmune disease, no use of immunosuppressants or steroids, and no hospitalization within the last 12 months. The healthy individual samples used for B cell sorting were collected through the NIHR Cambridge BioResource.<br><br>Patients with CD (blood)<br>Patients with active Crohn's disease were recruited from a specialist IBD clinic at Addenbrooke's Hospital, before starting treatment. Diagnosis was made using standard endoscopic, histological and radiological criteria. All patients had at least moderately active Crohn's disease at enrolment as evidenced by clinical symptoms in conjunction with some or all of elevated C-reactive protein, elevated fecal calprotectin, radiologically active disease or endoscopically active disease.<br><br>Patients with CD (MSLN)<br>Patients with active Crohn's disease were recruited from the Medical University of Vienna who were undergoing bowel resection for active disease. LNs close to areas of inflammation, which had been resected intra-operatively were obtained.<br><br>Patients with CD ((gut mucosa)<br>Patients with active Crohn's disease were recruited from the Stanford Hospital who were undergoing bowel resection for active disease.
Ethics oversight	Ethical approval was obtained from the Medical University of Vienna's Institutional Review Board (EK number: 1480/2016), from the Cambridgeshire Regional Ethics committee (REC08/H0306/21, REC08/H0308/176) and) and from Stanford University (Stanford Inflammatory Bowel Disease Immune Repertoire IRB 64710). All patients provided written informed consent.

Note that full information on the approval of the study protocol must also be provided in the manuscript.

Field-specific reporting

Please select the one below that is the best fit for your research. If you are not sure, read the appropriate sections before making your selection.

☒ Life sciences ☐ Behavioural & social sciences ☐ Ecological, evolutionary & environmental sciences

For a reference copy of the document with all sections, see nature.com/documents/nr-reporting-summary-flat.pdf

Life sciences study design

All studies must disclose on these points even when the disclosure is negative.

Sample size	Sample sizes were determined such that each disease group contained >=8 patients, which we have previously shown to be sufficient to distinguish during active immune responses, such as in early HIV infection (Hoehn et al 2015).
Data exclusions	Samples with <1000 unique BCRs from the sequencing were excluded.

Replication	We have previously shown that technical replicates using these methods are highly correlated (Petrova et al 2018) and therefore replicates were not performed on an individual patient basis.
Randomization	NA. Patients selected on the basis of no/limited prior treatment.
Blinding	During sample processing and sequencing.

Reporting for specific materials, systems and methods

We require information from authors about some types of materials, experimental systems and methods used in many studies. Here, indicate whether each material, system or method listed is relevant to your study. If you are not sure if a list item applies to your research, read the appropriate section before selecting a response.

Materials & experimental systems

n/a	Involved in the study	
☐	☒	Antibodies
☒	☐	Eukaryotic cell lines
☒	☐	Palaeontology and archaeology
☒	☐	Animals and other organisms
☒	☐	Clinical data
☒	☐	Dual use research of concern
☒	☐	Plants

Methods

n/a	Involved in the study	
☒	☐	ChIP-seq
☐	☒	Flow cytometry
☒	☐	MRI-based neuroimaging

Antibodies

Antibodies used	CD3 BV711 Biolegend Cat # 317328<br>CD19 BV785 Biolegend Cat # 302240<br>CD27 PE-Cy7 eBiosciences Cat# 25-0279<br>CD56 FITC Biolegend Cat# 304605<br>IgD FITC BD Cat# 555778<br>CD24 PerCP-Cy5.5 BD Cat # 561647<br>CD38 BV711 Biolegend 353426
Validation	See manufacturers' notes

Plants

Seed stocks	*Report on the source of all seed stocks or other plant material used. If applicable, state the seed stock centre and catalogue number. If plant specimens were collected from the field, describe the collection location, date and sampling procedures.*
Novel plant genotypes	*Describe the methods by which all novel plant genotypes were produced. This includes those generated by transgenic approaches, gene editing, chemical/radiation-based mutagenesis and hybridization. For transgenic lines, describe the transformation method, the number of independent lines analyzed and the generation upon which experiments were performed. For gene-edited lines, describe the editor used, the endogenous sequence targeted for editing, the targeting guide RNA sequence (if applicable) and how the editor was applied.*
Authentication	*Describe any authentication procedures for each seed stock used or novel genotype generated. Describe any experiments used to assess the effect of a mutation and, where applicable, how potential secondary effects (e.g. second site T-DNA insertions, mosiacism, off-target gene editing) were examined.*

Flow Cytometry

Plots

Confirm that:

☒ The axis labels state the marker and fluorochrome used (e.g. CD4-FITC).

☒ The axis scales are clearly visible. Include numbers along axes only for bottom left plot of group (a 'group' is an analysis of identical markers).

☐ All plots are contour plots with outliers or pseudocolor plots.

☒ A numerical value for number of cells or percentage (with statistics) is provided.

Methodology

Sample preparation	Each participant provided 100 mL of peripheral venous blood collected into 9 mL sodium citrate tube. Peripheral blood mononuclear cells (PBMCs) were isolated using Leucosep tubes (Greiner Bio-One) with Histopaque 1077 (Sigma) by centrifugation at 800x g for 15 min at room temperature. PBMCs at the interface were collected, rinsed twice with autoMACS running buffer (Miltenyi Biotech) and cryopreserved in FBS with 10% DMSO. All samples were processed within 4 h of collection. Samples were stored at 4°C and acquired within 4 h using a 5-laser BD Symphony X-50 flow cytometer. Single color compensation tubes (BD CompBeads) or cells were prepared for each of the fluorophores used and acquired at the start of each flow cytometer run. For direct enumeration of B cells, an aliquot of whole blood (50 µl) was added to BD TruCount tubes with 20µl- BD Multitest 6-color TBNK reagent (BD Biosciences) and processed as per the manufacturer's instructions. Samples were gated in FlowJo v10.2 using the following B cells markers Naive (CD19+IgD+CD27−), double-negative B cells (DN) (CD19+IgD−CD27−), non-switched(NS)memory (CD19+CD27+CD24+CD38+), memory (switched) (CD19+IgD−CD27 +CD24+CD38+) and plasmablasts (CD19−CD20−CD27+CD24+CD27+CD38+). The number of cells falling within each gate was recorded. Plasmablasts were sorted (CD19+CD38+IgD-CD27+CD24-) fresh into lysis buffer and then the lysed cells frozen.
Instrument	BD Influx lasers/colours 4/16 jet-in-air
Software	FlowJo v10.2
Cell population abundance	Post-sort purity check (>95%), as well as through assessment of isotype usages within the sorted populations from the sequencing data.
Gating strategy	Total lymphocytes were gated into live -dead. From live, CD3- and CD3+ cells were separated. CD3- cells were separated into CD14- and CD14+. From CD14-, CD19+ memory cells were gated. Naive cells were gated (CD19+IgD+CD27−), double-negative B cells (CD19+IgD−CD27−), non-switched memory (CD19+CD27+CD24+CD38+), memory (switched) (CD19+IgD −CD27+CD24+CD38+) and plasmablasts (CD19−CD20−CD27+CD24+CD27+CD38+). The number of cells falling within each gate was recorded. Plasmablasts were sorted (CD19+CD38+IgD-CD27+CD24-)

☒ Tick this box to confirm that a figure exemplifying the gating strategy is provided in the Supplementary Information.
